# Reduction of Conduction Velocity in Patients with Atrial Fibrillation

**DOI:** 10.3390/jcm10122614

**Published:** 2021-06-14

**Authors:** Annejet Heida, Mathijs S. van Schie, Willemijn F. B. van der Does, Yannick J. H. J. Taverne, Ad J. J. C. Bogers, Natasja M. S. de Groot

**Affiliations:** 1 Erasmus Medical Center, Department of Cardiology, 3015 GD Rotterdam, The Netherlands; a.heida@erasmusmc.nl (A.H.); m.vanschie@erasmusmc.nl (M.S.v.S.); w.vanderdoes@erasmusmc.nl (W.F.B.v.d.D.); 2 Erasmus Medical Center, Department of Cardiothoracic Surgery, 3015 GD Rotterdam, The Netherlands; y.j.h.j.taverne@erasmusmc.nl (Y.J.H.J.T.); a.j.j.c.bogers@erasmusmc.nl (A.J.J.C.B.)

**Keywords:** atrial fibrillation, electrophysiology, atrial remodeling, conduction velocity

## Abstract

It is unknown to what extent atrial fibrillation (AF) episodes affect intra-atrial conduction velocity (CV) and whether regional differences in local CV heterogeneities exist during sinus rhythm. This case-control study aims to compare CV assessed throughout both atria between patients with and without AF. Patients (*n* = 34) underwent intra-operative epicardial mapping of the right atrium (RA), Bachmann’s bundle (BB), left atrium (LA) and pulmonary vein area (PVA). CV vectors were constructed to calculate median CV in addition to total activation times (TAT) and unipolar voltages. Biatrial median CV did not differ between patients with and without AF (90 ± 8 cm/s vs. 92 ± 6 cm/s, *p* = 0.56); only BB showed a CV reduction in the AF group (79 ± 12 cm/s vs. 88 ± 11 cm/s, *p* = 0.02). In patients without AF, there was no predilection site for the lowest CV (P_5_) (RA: 12%; BB: 29%; LA: 29%; PVA: 29%). In patients with AF, lowest CV was most often measured at BB (53%) and ranged between 15 to 22 cm/s (median: 20 cm/s). Lowest CVs were also measured at the LA (18%) and PVA (29%), but not at the RA. AF was associated with a prolonged TAT (*p* = 0.03) and decreased voltages (P_5_) at BB (*p* = 0.02). BB was a predilection site for slowing of conduction in patients with AF. Prolonged TAT and decreased voltages were also found at this site. The next step will be to determine the relevance of a reduced CV at BB in relation to AF development and maintenance.

## 1. Introduction

Intra-atrial conduction velocity (CV) is determined by ion channel properties, cell-to-cell coupling, wavefront geometry and muscle thickness [1,2]. Areas of reduced CV are associated with initiation and perpetuation of atrial fibrillation (AF) [3]. In 12 patients with ischemic heart disease or Wolff–Parkinson–White syndrome who underwent cardiac surgery, the average CV measured during sinus rhythm (SR) at the right atrial free wall in an area of 3 × 4 cm^2^ was 88 cm/s [4]. A comparable average CV of 89 ± 13 cm/s was found at Bachmann’s Bundle (BB) in 185 patients undergoing coronary artery bypass surgery [5].

There is only one report on comparison of CV during SR between patients with and without AF [6]. In this endocardial mapping study, paroxysmal AF was associated with a reduction of CV to 60 ± 12 cm/s at the right atrium (RA) and to 51 ± 11 cm/s at the left atrium (LA) compared to patients with atrioventricular nodal re-entrant tachycardia and Wolff–Parkinson–White syndrome, respectively (RA: 83 ± 13 cm/s and LA: 70 ± 10 cm/s) [6]. Additionally, the basal, septal and annular regions of the RA showed a reduction in CV in patients with paroxysmal AF (basal region: 54 ± 19 cm/s vs. 82 ± 25 cm/s; septal region: 64 ± 20 cm/s vs. 93 ± 32 cm/s; annular region: 60 ± 12 cm/s vs. 83 ± 13 cm/s, all *p* < 0.05) [6]. However, mapping was performed at only a limited number of sites at the RA and LA. Remarkably, the CV in this control group was much lower than CVs assessed in the studies described above.

To date, it is unknown to what extent AF episodes affect intra-atrial CV and whether there are regional differences in local CV during SR at the RA, BB and the LA including the pulmonary vein area (PVA). The aim of our case-control study is therefore to compare CV assessed at a high-resolution scale throughout both atria between patients with AF and without atrial tachyarrhythmias and to investigate which region is most affected by AF episodes.

## 2. Materials and Methods

### 2.1. Study Population and Setting

The study population consisted of participants undergoing elective open-heart surgery in the Erasmus Medical Center. Indications for elective cardiac surgery were either coronary artery disease, aortic valve- or mitral valve disease, or a combination of these. The case group consisted of patients with a history of documented AF. All SR recordings in this group were made after electrical cardioversion. The control group consisted of patients without atrial tachyarrhythmias. Participants were matched based on known confounders of intra-atrial conduction disorders, i.e., age [7], BMI [8] and left atrial enlargement [9]. Echocardiographic images were used to identify atrial dilatation. Participants were enrolled between March 2012 and April 2018. This study is approved by the institutional Medical Ethical Committee (resp. MEC 2010-054 [10] and MEC 2014-393 [11]). Written informed consent was obtained from all patients prior to the surgical procedure. The study complied with the Declaration of Helsinki. Clinical data were extracted from electronic patient files.

### 2.2. Mapping Procedure

**Figure 1:**
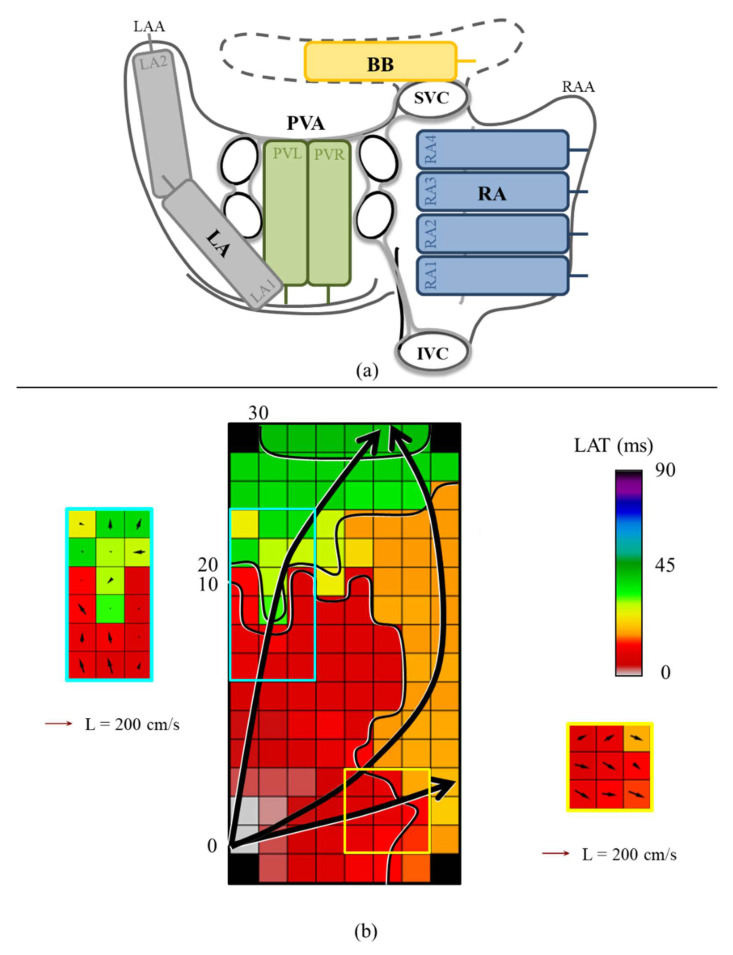
**Method of epicardial mapping.** (**a**) Mapping scheme on a schematic posterior view of the RA, BB, LA and PVA; (**b**) an example of a color-coded activation map with isochrones (black lines) drawn at 10 ms. The black arrow indicates the main wave direction. Examples of the corresponding CV map using discrete velocity vectors are shown next to the activation map. CV vectors are depicted per electrode. cm/s = centimeter/second; ms = milliseconds; BB = Bachmann’s Bundle; CT = conduction time; IVC = inferior vena cava; L = length; LA = left atrium; LAA = left atrial appendage; LAT = local activation time; PVA = pulmonary vein area; PVL = left pulmonary vein; PVR = right pulmonary vein; RA = right atrium; RAA = right atrial appendage; SVC = superior vena cava.

As previously described, high-resolution epicardial mapping was performed during open heart surgery [12]. A bipolar pacemaker wire was temporarily attached to the right atrial free wall to function as a reference electrode. A steel wire was fixed in the thoracic subcutaneous tissue serving as an indifferent electrode. Epicardial mapping was performed using an unipolar 128- or a 192-electrode array (electrode diameter respectively 0.65 and 0.45 mm, interelectrode distances of 2 mm) and following a predefined scheme (Figure 1A), covering the epicardial surface of the RA (from the inferior caval vein up to the right atrial appendage, perpendicular to the caval veins), PVA (from the sinus transversus, alongside the borders of the pulmonary veins towards the atrioventricular groove), LA (from the lower border of the left pulmonary vein along the left atrioventricular groove towards the left atrial appendage) and BB (from the tip of left atrial appendage behind the aorta towards the superior cavo-atrial junction).

At each site, five seconds of SR mapping were recorded, including unipolar epicardial electrograms, a surface electrocardiogram, a bipolar reference electrogram and a calibration signal (amplitude: 2 mV, duration: 1000 ms). Recordings were sampled with a rate of 1 kHz, amplified (gain: 1000), filtered (bandwidth: 0.5–400 Hz), analogue-to-digital-converted (16-bits) and stored on a hard disk.

### 2.3. Mapping Data Processing

The steepest negative slopes of all atrial potentials were automatically annotated using custom-made mapping software when the amplitude exceeded twice the signal-to-noise ratio [5,13]. For each electrode, the local activation time was determined and color-coded activation maps were reconstructed as illustrated in Figure 1b. All annotations were visually verified. Premature extrasystolic and aberrant beats were excluded from analysis, along with mapping sites in which less than 50% was annotated. Areas of simultaneous activation were excluded from analysis in order to avoid inclusion of far field potentials.

### 2.4. Calculation of Local Conduction Velocities

Local CV was computed as an average of velocity estimations between neighbouring electrodes (longitudinal, transversal and diagonal) using discrete velocity vectors as previously described by van Schie et al. [14]. Relative frequency distribution histograms of CVs were constructed to calculate median CV and variance of CV (Δ P_5_-P_95_). For identification of areas of ‘slow conduction’, the 5th percentiles of the relative frequency distribution of CVs were determined. Additionally, the total activation times (TAT) of both atria and for each mapping site separately was determined by relating the first and last local activation time to the reference electrode. To compare voltage characteristics between patients with and without AF, we measured the peak-to-peak amplitude of the steepest deflection of each unipolar potential to construct relative frequency histograms. For determination of ‘low voltage’, we calculated the 5th percentile of the relative frequency distribution histograms of the voltages of all unipolar potentials.

### 2.5. Statistical Analysis

Statistical analysis was performed with SPSS version 25 (IBM Corporation, Armonk, NY, USA). All data were tested for normality using Shapiro-Wilk test. Continuous normally distributed data were expressed as mean ± standard deviation and skewed data as median (interquartile range). To compare continuous parameters between the AF and no AF group, a paired samples t-test or Wilcoxon signed rank test was used. Categorical data is expressed as absolute numbers (percentages) and analyzed with (McNemar’s symmetry) χ2 or McNemar’s exact test, if applicable. A two-sided *p*-value of <0.05 was considered statistically significant.

## 3. Results

### 3.1. Study Population

As presented in Table 1, baseline characteristics between the AF group (*n* = 17, 73 ± 7 years; 11 (64.7%) male) and no AF group (*n* = 17, 74 ± 7 years; 9 (52.9%) male) did not differ (all *p* ≥ 0.05). Participants in the AF group either had paroxysmal AF (*n* = 6, 27.3%), persistent AF (*n* = 9, 40.9%) or longstanding persistent AF (*n* = 2, 9.1%). Patients in the AF group had 1 month (0.5–4.0) AF before electrical cardioversion.

### 3.2. Mapping Data Characteristics

In total, 164,099 potentials (9192 (7421–11,250) potentials/patient) in the AF group and 150,015 potentials (8533 (7392–10,699) potentials/patient) in the no AF group were analyzed (*p* = 0.23). Due to simultaneous activation, 2.4% of the potentials in the AF group and 2.5% of the potentials in the no AF group were excluded from analysis. SR cycle length during epicardial mapping was 808 ± 117 ms in the AF group and 881 ± 213 ms in the no AF group (*p* = 0.17).

### 3.3. Conduction Velocity Throughout Both Atria

**Figure 2:**
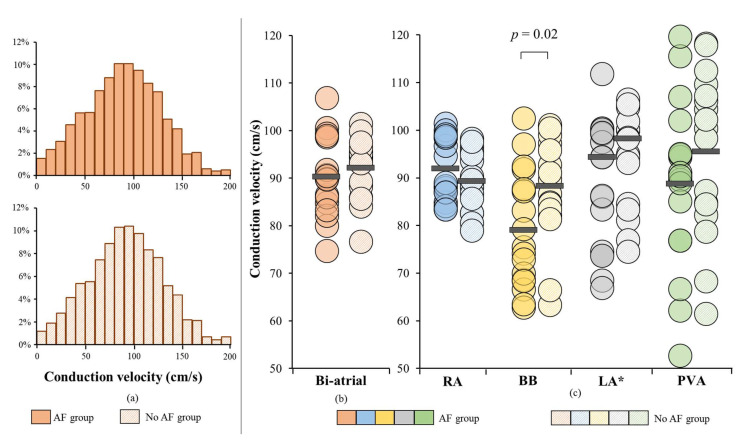
**CV in patients with and without a history of AF.** (**a**) biatrial CV histograms of patients with and without a history of AF; (**b**) biatrial median CV displayed for each patient (**c**) median CV displayed for each patient per region separately. *Non-normally distributed. cm/s = centimeter/second; CV = conduction velocity; AF = atrial fibrillation; BB = Bachmann’s Bundle; LA = left atrium; PVA = pulmonary vein area; RA = right atrium.

Figure 2A shows histograms of the relative frequency distribution of CVs throughout both atria for the AF group and no AF group separately. As can be seen, the CV histograms of both groups are comparable. Furthermore, Figure 2 demonstrates the median CV in the AF and no AF group for both atria (b) and for each location separately (c).

Biatrial median CV in the no AF group ranged from 77 to 107 cm/s and in the AF group from 75 to 101 cm/s. There was no difference in biatrial median CV between both groups (AF: 90 ± 8 cm/s vs. no AF: 92 ± 6 cm/s, *p* = 0.56, Figure 2b). Additionally, variation of CV was also comparable between the AF and no AF group (Δ P_5_-P_95_: 129 ± 8 ms vs. 129 ± 10 ms, *p* = 0.88).

### 3.4. AF-related Reduction of Conduction Velocity

**Figure 3:**
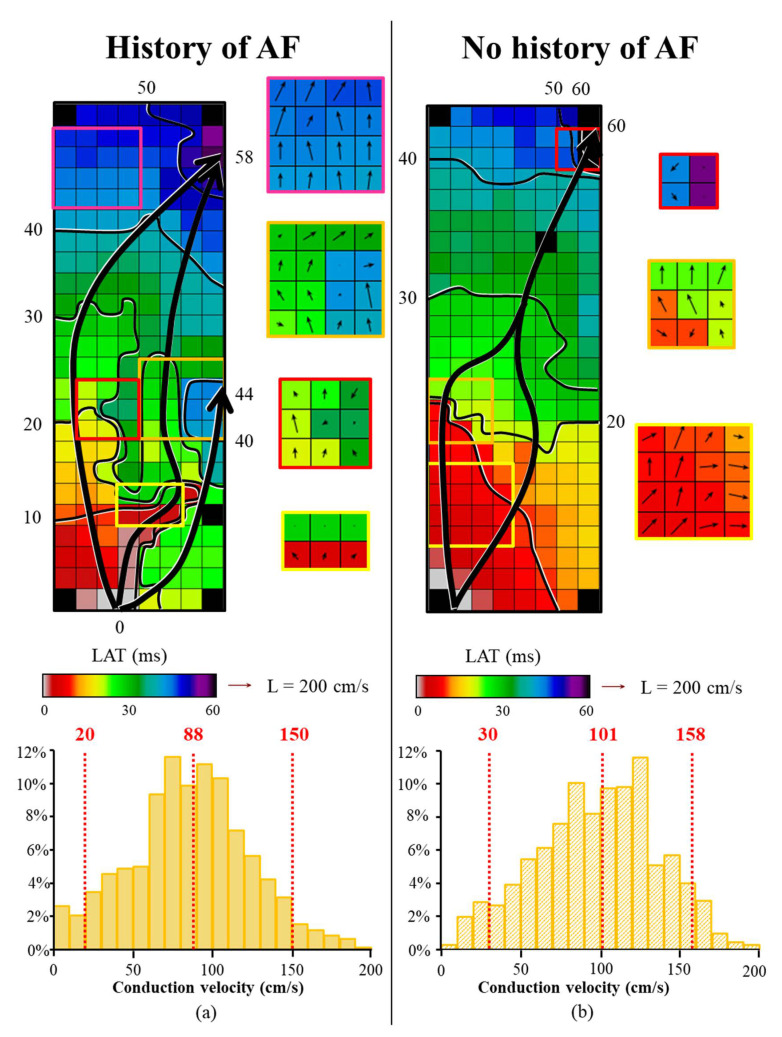
**Color-coded activation maps and corresponding CV histograms at BB of a patient with and without a history of AF.** The upper panel shows two examples of a color-coded activation maps at BB obtained from a patient with a history of AF (**a**) and a patient without a history of AF (**b**). Corresponding CV maps are depicted next to the activation map. CV vectors are depicted per electrode. In the patient with AF, a large area of slowing of conduction is present in the lower part of the activation map, represented by crowding of isochrones. The colored boxes indicate smooth wavefront propagation (pink rectangle) and local conduction heterogeneities (yellow, red and orange rectangles). In the patient without AF, the sinus rhythm wavefront encounters only some small areas of conduction delay (orange and red rectangles). Isochrones (black lines) drawn at 10 ms. The black arrows indicates the main wave direction. Lower panel: corresponding CV histograms of the patient with AF (**a**) and without AF (**b**) recorded at BB. The dashed lines represent the 5th, 50th and 95th percentiles of the relative frequency distribution of CVs. cm/s = centimeter/second; ms = milliseconds; BB = Bachmann’s bundle; CV = conduction velocity; LAT = local activation time.

Figure 3 illustrates two examples of color-coded activation maps at BB obtained from a patient with a history of AF (a) and a patient without a history of AF (b). Corresponding CV maps are depicted next to the activation map. In the control patient, the SR wavefront encounters only some small areas of conduction delay, represented by crowding of isochrones in the left middle part of the array and right upper corner, resulting in a median CV of 101 cm/s. However, in the patient with a history of AF, a large area of slowing of conduction is present in the lower part of the activation map, resulting in a lower median CV of 88 cm/s. The lower panel of Figure 3 illustrates the corresponding relative frequency distribution of CVs at BB of the same patients.

**Figure 4:**
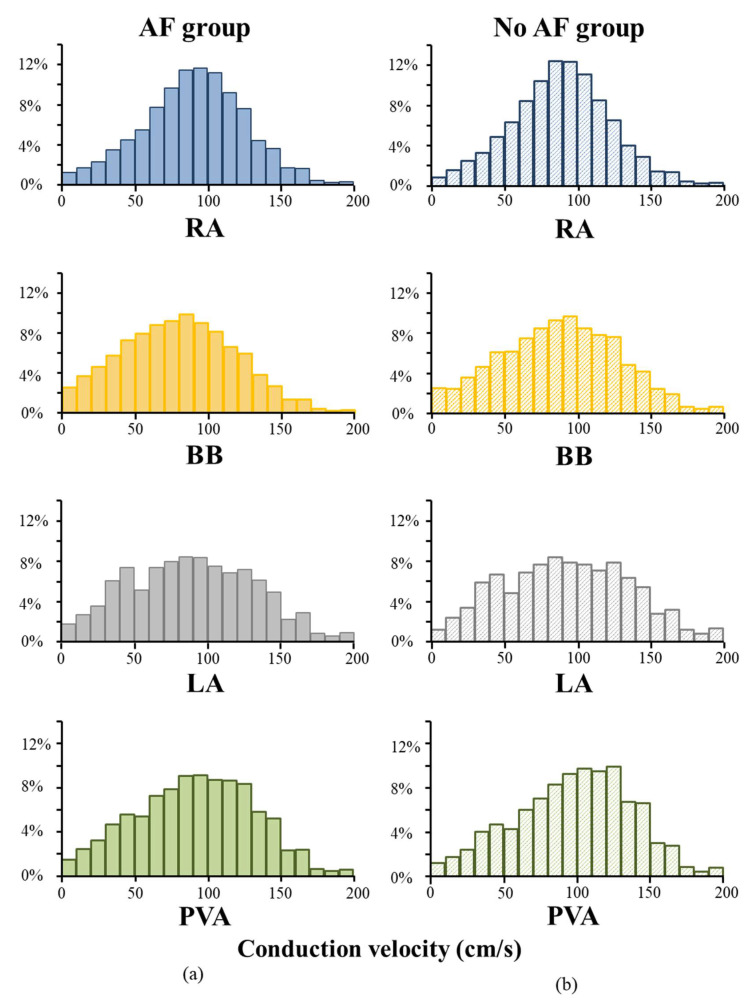
**CV histograms of the AF and no AF group.** Relative frequency distributions of CVs in the AF (**a**) and no AF group (**b**) shown for each location separately. cm/s = centimeter/second; AF = atrial fibrillation; BB = Bachmann’s Bundle; LA = left atrium; PVA = pulmonary vein area; RA = right atrium.

Figure 4 shows relative frequency distributions of CVs in the entire AF and no AF group for each location separately. Compared to patients without AF, slowing of conduction was solely found at BB in patients with AF (BB: 79 ± 12 cm/s vs. 88 ± 11 cm/s, *p* = 0.02; RA: 92 ± 7 cm/s vs. 89 ± 6 cm/s, *p* = 0.35; PVA: 89 ± 18 cm/s vs. 96 ± 17 cm/s, *p* = 0.32, LA: 94 cm/s (84–101) vs. 98 cm/s (84–101), *p* = 0.21) (Figure 2C). However, as shown in Table 2, the variance of CV per location, including BB, was comparable between patients in the AF and no AF group (all *p* ≥ 0.05).

**Figure 5:**
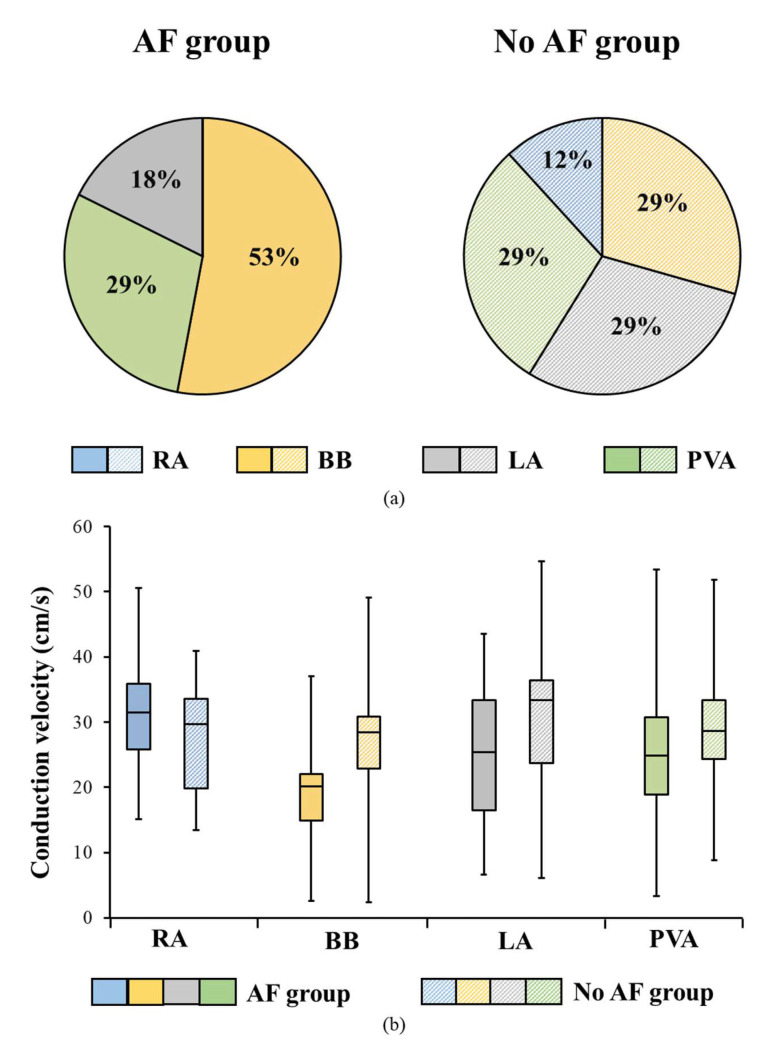
**Predilection site for the lowest CV** (**a**) Location of the lowest CV (defined as the 5th percentile of the relative frequency distribution of CV), within each AF patient (left) and no AF patient (right); (**b**) Distribution of the lowest CVs shown for each location separately. cm/s = centimeter/second; AF = atrial fibrillation; BB = Bachmann’s Bundle; LA = left atrium; PVA = pulmonary vein area; RA = right atrium.

Figure 5a shows for the AF and no AF group at which region the lowest CV (5th percentile of the CV histogram) within each patient occurred. In patients without AF, there was no predilection site for the lowest CVs (P_5_) (RA: 12%, *n* = 2; BB: 29%, *n* = 5; LA: 29%, *n* = 5; PVA: 29%, *n* = 5). In patients with AF, lowest CV was most often measured at BB (53%, *n* = 9) and were also measured at the LA (18%, *n* = 3) and PVA (29%, *n* = 5), but not at the RA (RA: 0%, *n* = 0). Figure 5b shows the distribution of the lowest CV for each location separately. At BB, the lowest CV (interquartile range) ranged between 15 and 22 cm/s (median 20 cm/s), while in the no AF group it ranged between 23 and 31 cm/s (median: 28 cm/s). At the RA, LA and PVA, lowest CVs are comparable between the AF and no AF group.

### 3.5. Relation between Conduction Heterogeneity and Total Activation Time

**Figure 6:**
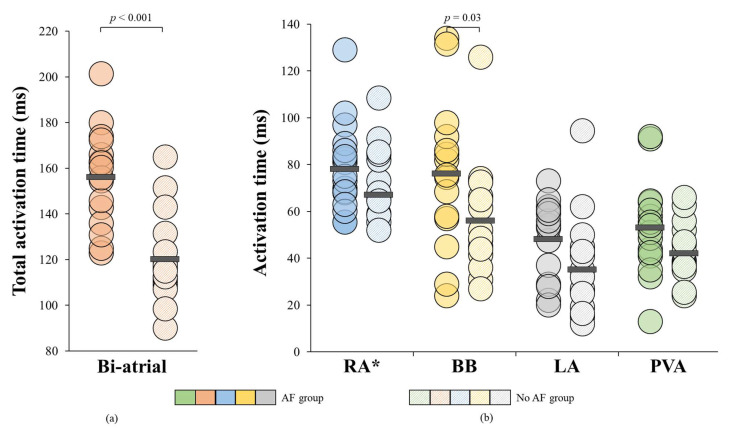
**TAT displayed for each individual patient for the entire atria and per region separately** (**a**) Biatrial TAT displayed for each individual patient; (**b**) TAT displayed for each patient per region separately. * Non-normally distributed. ms = milliseconds; BB = Bachmann’s Bundle; LA = left atrium; PVA = pulmonary vein area; TAT = total activation time; RA = right atrium.

Figure 6 illustrates for each patient the TAT (a) and the TAT per region separately (b). AF was associated with a prolonged TAT (156 ± 21 ms vs. 120 ± 22 ms, *p* < 0.001) and TAT was particularly prolonged at BB (76 ± 31 ms vs. 58 ± 23 ms, *p* = 0.03).

### 3.6. Atrial Fibrillation Episodes and Unipolar Voltages

The lowest voltages (5th percentiles of the voltage histogram) differed between the AF and no AF group only at BB (Table 3). Patients in the AF group had a low voltage of 0.9 ± 0.6 mV at this location, whereas in the control group it was 1.5 ± 0.9 mV (*p* = 0.02). The lowest voltages did not differ between both groups at RA, PVA and LA (all *p* ≥ 0.05).

## 4. Discussion

### 4.1. Key Findings

This high-resolution intraoperative mapping study is the first to explore the association between CV and a history of AF in both atria. CV was reduced in the AF group only at BB. However, the variance of CV was comparable between both groups at this site. BB was also a predilection site for slowing of conduction in the AF group, as the lowest CVs were mostly located at this site. In patients without AF, the lowest CVs were found at all locations. Moreover, TAT was prolonged and voltages were decreased at BB in patients with AF. AF was not associated with a reduced CV, a prolonged TAT or lower voltages at the RA, PVA and LA.

### 4.2. Reduced Conduction Velocity as a Prerequisite for AF Onset

AF is the most common arrhythmia and induces both electrical and structural remodeling [15,16]. Longer duration of AF increases the degree of structural and electrical remodeling [17,18]. Electrical remodeling consists of alterations in ion channel expression and is reversible within one week of SR [16,19,20,21,22,23,24]. Structural remodeling consists of atrial fibrosis and side-to-side cell uncoupling and is still or partially present after 4 months of SR [24,25,26,27]. Both remodeling processes affect intra-atrial CV and may explain the increased vulnerability to AF. In the present study, we measure immediately after ECV, which means that both electrical and structural remodeling are present. After AF termination, it is generally assumed that the combination of increased dispersion of the refractory period and a reduced CV in combination with the presence of triggers such as premature beats may increase the susceptibility to AF recurrence.

As previously mentioned, the average CV measured during SR at the right atrial free wall in patients without atrial remodeling is 88 ± 9 cm/s [4]. A similar CV of 89 ± 13 cm/s was found at BB in 185 patients undergoing coronary artery bypass surgery, of whom only 13 had paroxysmal AF [5]. Comparing these CVs to the CV in our no AF group, our results are comparable at RA and BB with a median CV of 89 ± 6 cm/s and 88 ± 11 cm/s, respectively.

In our AF group, CV was reduced and TAT was prolonged only at BB compared to patients without AF. Previously, we determined the most accurate methodology to measure local conduction heterogeneity and, as a sub-analysis, we determined the median CV at all locations in patients with paroxysmal AF and no AF [14]. Subsequently, we also found a reduced CV at BB in patients without AF [14]. However, in that study we did not include patients with persistent or longstanding persistent AF who have varying degrees of atrial remodeling. In addition, confounders of intra-atrial conduction, such as age, body mass index and left atrial enlargement, were not considered [7,8,9]. Patients were significantly older in the paroxysmal AF group (71 ± 9 years vs. 66 ± 10 years, *p* < 0.001) and more patients had left atrial enlargement (41% vs. 19%, <0.001) [14]. Another study, performed by Zheng et al., compared the average CV at the RA and LA between patients with paroxysmal AF and control patients with respectively atrioventricular nodal re-entry tachycardia and Wolff-Parkinson White syndrome using three-dimensional endocardial mapping [6]. In that study, a history of AF was associated with a reduced CV at both locations which was more pronounced at the LA than the RA [6]. Remarkably, CV was much lower than our findings in the AF group (LA: 50 cm/s vs. 94 cm/s; RA: 60 cm/s vs. 92 cm/s). To estimate CV, they only compared a triplet of sites, when we computed, for each atrial potential, an average of velocity estimations between all eight adjacent electrodes [6,14]. Furthermore, those triplet numbers of locations were not measured simultaneously during the same beat, as we did in our study with the 128- or 192 electrode array [6]. It is likely that the CV measured in our study population is more accurate and therefore did not show a difference in CV at the RA and LA between patients with and without AF. In addition, no measurements were made of the PVA and BB and the study population was underpowered with only six paroxysmal AF patients for comparison [6].

We only evaluated regional differences in CV between patients with and without AF and not specific areas within each region. As previously mentioned, CV is determined, among other things, by wavefront geometry and muscle thickness [1,2]. The smooth, apparently uniform walls of the LA are composed of multiple layers of differently aligned myocardial fibers, with marked regional variations in thickness [28,29]. In the RA, muscular bundles are larger, e.g., the terminal crest, and as a result of the presence of pectinate muscles, the RA wall is not uniform in thickness [30]. In normal hearts, inhomogeneities are already present in both gross structure and myoarchitecture [30]. Future studies should investigate whether there is a relation between the magnitude of CV and specific anatomic features in patients with AF.

### 4.3. Bachmann’s Bundle as a Predilection Site for Atrial Fibrillation

The propagation of wavefronts occurs preferably and more rapidly along longitudinal cardiac fibers instead of perpendicular to them [31,32]. BB has parallel aligned muscle bundles, making it the preferred route for interatrial conduction [33]. BB is also believed to play an important role in the pathophysiology of AF [34,35]. Clinical studies have found an association between interatrial block and development of AF [34,36]. In our study, patients with AF had a reduced CV with TAT prolongation at this site. A histological examination of 10 hearts from patients with paroxysmal AF and 10 hearts from patients without AF showed that fibro-fatty tissue was much more extensive in patients with AF [37]. This can lead to a disruption of cell-to-cell connections, disturbed wavefront geometry and thus a reduced CV and prolonged TAT.

### 4.4. Low Voltages and Atrial Fibrillation

Low-voltage areas are a commonly used surrogate marker for the presence of atrial fibrosis, which plays a key role in the maintenance of AF [38]. Atrial fibrosis, in turn, affects local CV by forming ‘zig-zag’ like conduction paths, leading to anisotropy and thus a reduced CV [1]. In the present study, we did not correlate low-voltage areas with CV, as it remains very challenging to identify a correct threshold to define low-voltage areas. Additionally, prior high-resolution epicardial mapping studies demonstrated no clear relationship between CV and low voltage areas [14,39]. In our study population, in general we have found lower voltages at BB in patients with AF compared to patients without AF.

### 4.5. Study Limitations

Patients in the AF group may have had varying degrees of atrial remodeling as some patients had persistent or longstanding persistent AF, while other patients had paroxysmal AF. In addition, in the AF group, SR recordings were made immediately after ECV, resulting in a different degree of electrical remodeling. Furthermore, this is a single center observational study with a small amount of patients. There is a risk of selection bias. Moreover, at different sinus rates, different exit pathways occur that can affect the CV. Direction-dependent CV heterogeneities could be missed as we only analyzed SR.

## 5. Conclusions

This high-resolution intraoperative mapping study is the first to examine the association between CV and a history of AF in both atria. At BB only, CV was reduced in the AF group. However, the variance of CV was comparable between both groups at this site. Additionally, BB was also a predilection site for slowing of conduction in the AF group, as the lowest CV was mostly located at this site. Moreover, the TAT was prolonged and voltages were decreased at BB in patients with AF. The next step will be to determine the relevance of a reduced CV at BB in relation to AF development and maintenance.

## Figures and Tables

**Table 1:** Characteristics of Participants.

	AF Group (*n* = 17)	No AF Group (*n* = 17)	*p* Value
Age-Years (mean ± SD)	73 ± 7	74 ± 7	0.60
Male Sex-*n* (%)	11 (64.7)	9 (52.9)	0.69
BMI-kg/m^2^ (Median (IQR))	24.9 (23.0–29.2)	25.3 (22.9–29.1)	0.16
Underlying Heart Disease-*n* (%)			1.00
IHD	2 (11.8)	2 (11.8)	
(i)VHD	15 (88.2)	15 (88.2)	
AVD	2 (11.8)	3 (17.6)	
AVD and CAD	2 (11.8)	2 (11.8)	
MVD	9 (52.9)	7 (41.2)	
MVD and CAD	2 (11.8)	3 (17.6)	
Echocardiography			
LVF-n (%)			1.00
Normal	11 (64.7)	12 (70.6)	
Mild dysfunction	3 (17.6)	2 (11.8)	
Moderate dysfunction	3 (17.6)	3 (17.6)	
Severe dysfunction	0 (0.0)	0 (0.0)	
Dilated LA >45 mm-*n* (%)	11/13 (84.6)	9/15 (60.0)	0.41
Medication-*n* (%)			
Antiarrhythmic Drugs			0.45
Class I	0 (0.0)	0 (0.0)	
Class II	11 (64.7)	7 (41.2)	
Class III	4 (23.5)	0 (0.0)	
Class IV	1 (5.9)	1 (5.9)	
Digoxin	5 (29.4)	0 (0.0)	0.06

*n* = number; SD = standard deviation; AF = atrial fibrillation; AVD = aortic valve disease; BMI = Body Mass Index; CAD = coronary artery disease; IHD = ischemic heart disease; LA = left atrium; LVF = left ventricular function; MVD = mitral valve disease; (i)VHD = (ischemic and) valvular heart disease.

**Table 2:** Variation of CV.

	AF Group (*n* = 17)	No AF Group (*n* = 17)	*p* Value
Variation of CV (Δ P_5_-P_95_)			
RA-cm/s (mean ± SD)	118 ± 9	114 ± 8	0.14
BB –cm/s (median (IQR))	121 (114–133)	129 (119–137)	0.31
PVA-cm/s (mean ± SD)	129 ± 12	130 ± 21	0.87
LA-cm/s (mean ± SD)	139 ± 11	143 ± 13	0.42

cm/s = centimeters/second; IQR = interquartile range; SD = standard deviation; AF = atrial fibrillation; BB = Bachmann’s bundle; LA = left atrium; PVA = pulmonary vein area; RA = right atrium.

**Table 3:** The lowest unipolar voltages presented for the AF and no AF group.

	AF Group (*n* = 17)	No AF Group (*n* = 17)	*p* Value
5th percentiles of unipolar voltages			
Bi-atrial-mV (median (IQR))	0.6 (0.6–1.0)	0.8 (0.6–1.1)	0.44
RA-mV (median (IQR))	1.1 ± 0.6	0.9 ± 0.3	0.20
BB-mV (mean ± SD)	0.9 ± 0.6	1.5 ± 0.9	0.02
PVA-mV (mean ± SD)	0.6 (0.5–1.4)	1.4 (0.6–2.7)	0.08
LA-mV (mean ± SD)	1.2 ± 0.7	1.2 ± 0.7	0.83

IQR = interquartile range; SD = standard deviation; AF = history of AF; BB = Bachmann’s bundle; LA = left atrium; PVA = pulmonary vein area; RA = right atrium.
